# ZeGlobalTox: An Innovative Approach to Address Organ Drug Toxicity Using Zebrafish

**DOI:** 10.3390/ijms18040864

**Published:** 2017-04-19

**Authors:** Carles Cornet, Simone Calzolari, Rafael Miñana-Prieto, Sylvia Dyballa, Els van Doornmalen, Helma Rutjes, Thierry Savy, Davide D’Amico, Javier Terriente

**Affiliations:** 1 ZeClinics SL, PRBB (Barcelona Biomedical Research Park), 08003 Barcelona, Spain; carles.cornet@zeclinics.com (C.C.); simone.calzolari@zeclinics.com (S.C.); rafael.minana@zeclinics.com (R.M.-P.); sylvia.dyballa@zeclinics.com (S.D.); 2 Pivot Park Screening Centre (PPSC), Kloosterstraat 9, 5349AB OSS, The Netherland; els.vandoornmalen@ppscreeningcentre.com (E.v.D.); helma.rutjes@ppscreeningcentre.com (H.R.); 3 Multilevel Dynamics in Morphogenesis Unit, USR3695 CNRS, 91190 Gif sur Yvette, France; thierry.savy@polytechnique.edu

**Keywords:** ZeGlobalTox, zebrafish, high-throughput, adverse drug reaction, drug toxicity, cardiotoxicity, neurotoxicity, hepatotoxicity

## Abstract

Toxicity is one of the major attrition causes during the drug development process. In that line, cardio-, neuro-, and hepatotoxicities are among the main reasons behind the retirement of drugs in clinical phases and post market withdrawal. Zebrafish exploitation in high-throughput drug screening is becoming an important tool to assess the toxicity and efficacy of novel drugs. This animal model has, from early developmental stages, fully functional organs from a physiological point of view. Thus, drug-induced organ-toxicity can be detected in larval stages, allowing a high predictive power on possible human drug-induced liabilities. Hence, zebrafish can bridge the gap between preclinical in vitro safety assays and rodent models in a fast and cost-effective manner. ZeGlobalTox is an innovative assay that sequentially integrates in vivo cardio-, neuro-, and hepatotoxicity assessment in the same animal, thus impacting strongly in the 3Rs principles. It Reduces, by up to a third, the number of animals required to assess toxicity in those organs. It Refines the drug toxicity evaluation through novel physiological parameters. Finally, it might allow the Replacement of classical species, such as rodents and larger mammals, thanks to its high predictivity (Specificity: 89%, Sensitivity: 68% and Accuracy: 78%).

## 1. Introduction

The direct costs of bringing a new drug to the market are continuously increasing. Nowadays, the estimated costs are higher than US $1 billion per drug, and most of that is spent in the clinical phases [1]. On the other hand, the pharmaceutical industry has multiplied its investments in R&D. However, this increase does not correlate well with an increased success rate in marketing new drugs. This is partly due to the high rate of compound failure during clinical trials, where only around 10% of the molecules entering phase 1 clinical trials are ultimately approved by the United States Food and Drug Administration (FDA) [2,3]. Lack of efficacy or drug safety (toxicity) are the major factors in drug attrition, with the lack of efficacy being the leading cause of drug attrition during clinical trials and unanticipated toxicity being the most common cause of post market withdrawal [3,4,5,6]. To reduce the large costs of drug development, and streamline the whole process, the need arises to identify potential adverse drug responses (ADR) as early as possible, and definitely before entering costly regulatory preclinical and clinical phases [7]. In that sense, toxicity affecting the liver, heart, and/or central nervous system (CNS) in humans are among the most common toxic effects induced by drugs [1,8,9,10].

During the drug discovery process (candidate selection and lead optimization), the traditional first step in safety understanding is to perform enzymatic or cell culture-based in vitro screenings [11]. These high-throughput assays require small compound quantities and reduce later animal testing, in line with the Replacement, Reduction and Refinement (3R) principle. Although useful as a first indication of putative drug toxicities (e.g., assays for interaction with the human Ether-a-go-go Related Gene (hERG) channel), they often have low predictions of the final human organ toxicity outcomes, which result from complex Absorption, Distribution, Metabolism and Excretion (ADME) mechanisms and cell and tissue interactions, which are biological processes that are difficult to mimic by in vitro approaches. On the other hand, mammalian toxicity studies remain the gold standard for risk prediction in humans, but they are highly expensive, time-consuming, require large amounts of test compounds, and they are not always predictive. Therefore, they are not suitable for early stage toxicology screenings of medium-large compound libraries.

In order to speed up the drug development pipeline, prioritize drug candidates for animal testing, and reduce unnecessary costs in later mammalian studies, academic and pharmaceutical industry researchers are showing an increasing interest in the zebrafish model. Given the high degree of conservation among species, the effects observed in zebrafish-based experiments are considered representative for other higher vertebrate species, including humans. Unlike in vitro models, zebrafish embryos represent a complex organism where metabolic pathways and other physiological reactions are already established and functional, allowing the evaluation of toxicity, while considering uptake, metabolic reactions, and excretion. Therefore, its use provides a closer scenario to human biology than in vitro systems. Moreover, zebrafish larvae provide several technical and economic advantages, due to its unique biological properties (extensively reviewed in [12,13]), for developing high-throughput drug screenings. Hence, their exploitation results in a reduction of time and cost, when compared with rodent studies, while providing higher informative value than in vitro studies [13,14,15]. Furthermore, according to international ethical regulations [16], zebrafish larvae up to 5 days post fertilization (dpf) are considered in vitro models and are accepted as an alternative to animal testing [17,18]. Therefore, their use is in accordance with the 3Rs principle.

**Figure 1:**
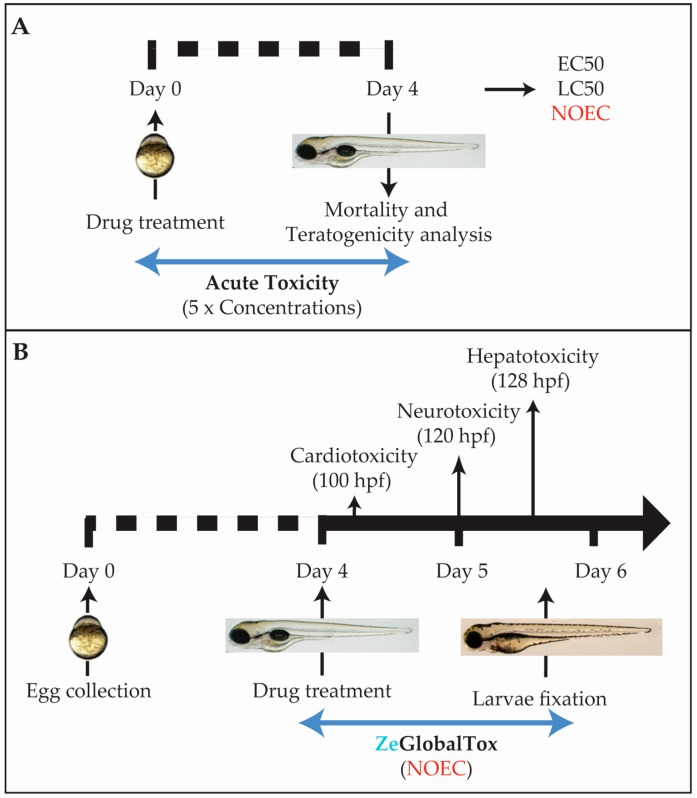
Complete ZeGlobalTox experimental setup. (**A**) Acute Toxicity experimental pipeline; (**B**) ZeGlobalTox experimental pipeline. Drugs are added from 96 hpf. Cardiotoxicity is evaluated at 100 hpf, neurotoxicity at 120 hpf, and hepatotoxicity at 132 hpf. Abbreviations: NOEC (no observed effect concentration).

Based on these facts, we have developed the ZeGlobalTox assay, an innovative experimental procedure that addresses organ-specific toxicity of different drugs on zebrafish larvae (up to 5 dpf). Thus, the proposed approach allows the independent analysis of cardio-, neuro-, and hepatotoxicity effects in the same animal (Figure 1A,B), as a proxy to predict their possible impact in human organs. This allows reducing the amount of larvae used, experimental time, and costs and quantity of the tested molecule. Furthermore, since the procedure uses whole animals, it has the advantage of addressing the tested compounds’ bioavailability, if necessary. We will show that by using ZeGlobaltox, a high toxicity predictivity is achieved—specificity (89%), sensitivity (68%), and accuracy (78%). These results reinforce the validation of zebrafish as a suitable model for pre-mammalian studies to reduce and/or replace mammalian vertebrate usage, experimental time, and cost during the process of drug discovery and development.

## 2. Results

### 2.1. Experimental Workframe

The ZeGlobalTox assay has been designed as a medium-throughput platform to detect, in the same animal, the three most concerning toxicities causing drug attrition, namely cardio-, neuro-, and hepatotoxicity. Several issues were considered when planning the experimental protocol. The main concern was that drug-induced mortality and/or developmental toxicity (teratogenicity) could mask possible organ-toxicities appearing later in development. To counteract this prospect, we included a preliminary Acute Toxicity assay performed with five logarithmic concentrations which follows the Organisation for Economic Cooperation and Development (OECD) guidelines (OECD 236) (Figure 1A). This preliminary assay allowed for the identification of non-mortal/non-teratogenic concentrations—no observed effect concentration (NOEC)—to use in the following assays. The hypothesis is that NOEC could affect organ physiology (organ toxicity), while uncoupled from putative developmental toxicity side-effects affecting organ development or function. Another consideration was to start drug incubations at 96 hours post fertilization (hpf), when the analysed organs are close to or already developed (Figure 1B). The overall aim is to understand the drug impact on organ physiology and function rather than early embryogenesis. The third consideration was to organize the sequential organ evaluation during the experimental and drug incubation time.

Among the three organs, the heart is developed earlier. In addition, cardiotoxic effects are observable shortly after compound incubation. Thus, cardiotoxicity evaluation was chosen first. Neurotoxicity was analysed second through the drug impact on motor behaviour (locomotion), which is a fundamental readout of CNS function. Although both autonomous swimming and liver development are completed by 5 dpf, phenotypes promoted by hepatotoxic effects require a longer drug exposure. In addition, part of the hepatotoxicity evaluation required fixed larvae. Then, hepatotoxicity was the final parameter evaluated. The integrated experimental pipeline is displayed in Figure 1.

### 2.2. Test Compounds

In order to validate our ZeGlobalTox platform, 24 compounds were evaluated; including four drugs used as positive toxic controls. To calculate the ZeGlobalTox predictive potential, compounds were chosen according to their known toxicity in humans, as displayed in known molecule toxicity databases such as TOXNET (Hazardous Substances Data Bank, HSDB), Side effects (EMBL), drugs.com, and ema.europa. The four selected control drugs have been selected due to their reported toxicities both in humans and zebrafish. Haloperidol, a known anti-dopaminergic antipsychotic drug, has been used as our positive cardiotoxic drug because it has been described to produce hERG blockade, QT interval prolongation, and arrhythmias both in humans and zebrafish [19,20,21]. MPTP, a prodrug to 1-methyl-4-phenylpyridinium (MPP^+^) first synthesized as an analgesic, has been shown to cause permanent Parkinson’s symptoms by destroying dopaminergic neurons in the substantia nigra [22]. Indeed, it has been used to model Parkinson’s disease in various animal models including zebrafish [23]. Hence, we used MPTP as the neurotoxic positive control drug. Finally, ethanol and acetaminophen (APAP, paracetamol) were used as our positive hepatotoxicity drugs. Both are well known molecules producing liver injury (extensively reviewed in [24,25]), with steatosis as a major side-effect for ethanol and liver malfunction and necrosis of liver tissue as the main toxic effect from paracetamol. Both hepatotoxic effects have also been reported in zebrafish larvae [26,27,28,29] (Table 1).

### 2.3. Acute Tox Analysis

**Figure 2:**
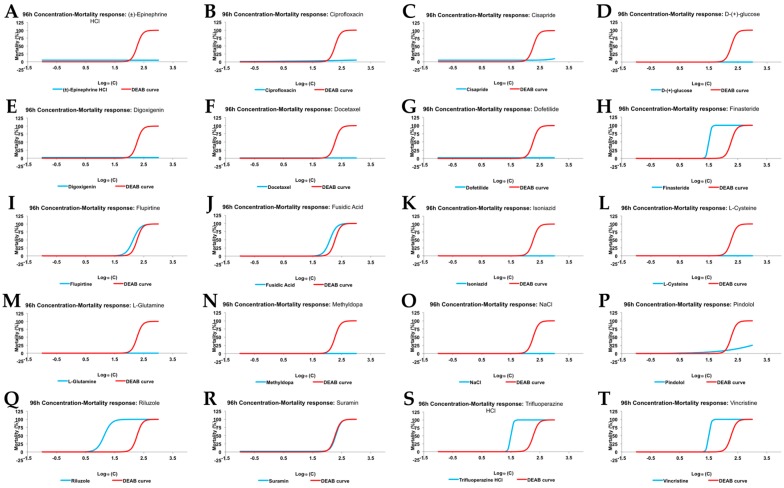
96 hpf mortality concentration response curve (red line), compared to DEAB (Diethylaminobenzaldehyde)(blue line), for (**A**) (±)-Epinephrine hydrochloride; (**B**) Ciprofloxacin; (**C**) Cisapride; (**D**) d-(+)-glucose; (**E**) Digoxigenin; (**F**) Docetaxel; (**G**) Dofetilide; (**H**) Finasteride; (**I**) Flupirtine; (**J**) Fusidic Acid; (**K**) Isoniazid; (**L**) l-Cysteine; (**M**) l-Glutamine; (**N**) Methyldopa; (**O**) NaCl; (**P**) Pindolol; (**Q**) Riluzole; (**R**) Suramin; (**S**) Trifluoperazine hydrochloride; and (**T**) Vincristine.

As explained above, the Acute Tox test was performed to determine the maximum drug concentration in which no mortality or gross teratogenic effects were observed (Non Observed Effect Concentration; NOEC). As a positive toxic drug we chose Diethylaminobenzaldehyde (DEAB), a Retinoid Acid inhibitor that promoted mortality and teratogenicity in a reproducible concentration dependent curve. 1% DMSO, which is the constant solvent concentration in all conditions, was used as the negative control. Mortality curves for all compounds at 96 hpf (blue line; Figure 2A–T), compared with DEAB curves (red line; Figure 2A–T), are shown in Figure 2. Additionally, the results from this analysis are displayed in Table 2.

### 2.4. Cardiotoxicity Analysis

Four cardiac parameters were evaluated to assess whether a compound was cardiotoxic: heart rate (beats per minute, BPM), QTc prolongation, ejection fraction (EJF), and cardiac arrest. Haloperidol has been described as cardiotoxic in humans and zebrafish [19] and was used as our cardiotoxic control. 1% DMSO was used as the negative control.

**Figure 3:**
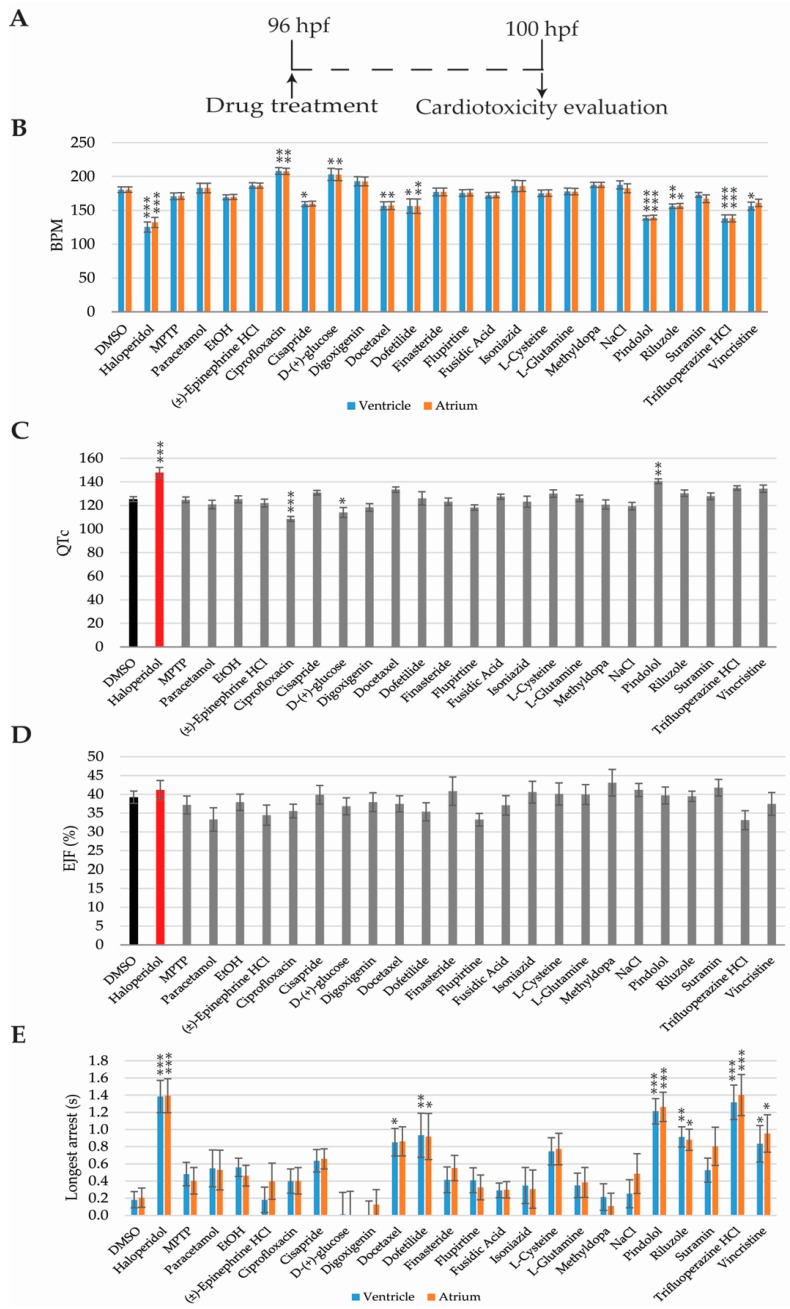
Cardiotoxicity evaluation results. (**A**) Scheme of the experimental procedure; (**B**) Bar graphs showing heart beat frequency in beats per minute (bpm); (**C**) QT corrected interval (QTc); (**D**) Ejection fraction (EJF); (**E**) and longest cardiac arrest of 100 h old zebrafish larvae. Asterisks indicate statistical significance after a One-way ANOVA: * *p* < 0.05; ** *p* < 0.01; *** *p* < 0.001. Black bar: negative control. Red bar: positive control. *n* = 16 but for DMSO *n* = 46.

Cardiotoxicity evaluation was performed from 96 hpf, when the zebrafish heartbeat is already stabilised [36,37], so the analysis might not be altered by unstable beating. Zebrafish hearts were video-recorded at 4 h after drug incubation (100 hpf) and analysed using the ZeCardio^®^ β software (Figure 3A).

Eight compounds—haloperidol, cisapride, docetaxel, dofetilide, pindolol, riluzole, trifluoperazine HCL, and vincristine—decreased the heart rate when compared to DMSO-only (Figure 3B). Longer cardiac arrest was promoted by the same compounds as well (Figure 3E). Inversely, zebrafish larvae treated with ciprofloxacin and d-(+)-glucose showed increased heart rates but no differences were observed in the duration of the cardiac arrest, when compared to the DMSO-only treated larvae (Figure 3B,E).

QTc interval prolongation was detected in larvae treated with haloperidol and pindolol, while ciprofloxacin and d-(+)-glucose showed a shorter QTc interval than the DMSO treated group (Figure 3C). Finally, no differences in ejection fraction were detected in any of the 24 compounds tested (Figure 3D).

### 2.5. Locomotor Activity Analysis

**Figure 4:**
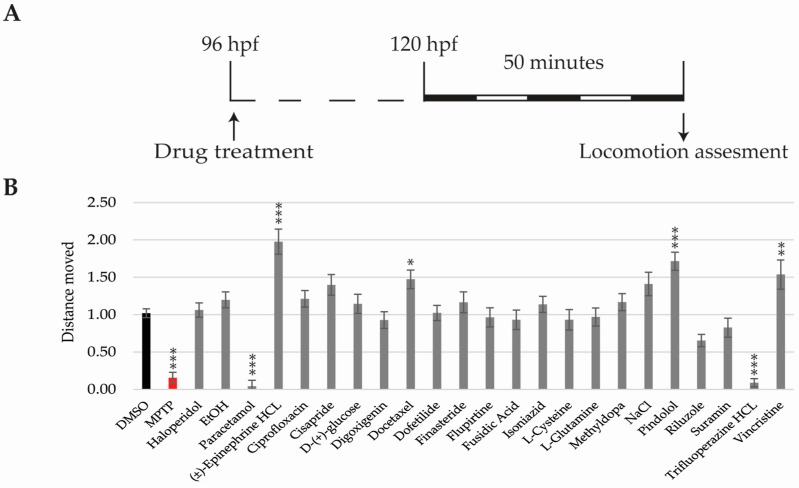
Locomotion results. (**A**) Scheme of the experimental procedure; (**B**) Bar graphs showing total distance moved corrected to the DMSO group. Asterisks indicate statistical significance after a One-way ANOVA * *p* < 0.05; ** *p* < 0.01; *** *p* < 0.001. Black bar: negative control. Red bar: positive control. Experiment performed once with 16 larvae per condition. *n* = 43 for the DMSO.

By 5 dpf, zebrafish larvae perform spontaneous swimming and their visual system is fully developed [31,38,39]. Therefore, behavioural experiments were performed from this time point (Figure 4A). Deviations in total distance moved, in response to photo-visual stimulation, were analysed as a direct measurement of neurotoxicity. Thus, drugs increasing or decreasing total distance moved when compared to the DMSO-only group were considered neurotoxic. As the positive neurotoxic control we used MPTP, which has been identified as a neurotoxic drug in humans and zebrafish [40].

Decreased motility was detected in MPTP, paracetamol, and trifluoperazine-HCL treated larvae, while (±)-epinephrine HCL, docetaxel, pindolol, and vincristine groups showed increased motility when compared to the DMSO (Figure 4B).

### 2.6. Hepatotoxicity Analysis

Zebrafish liver development is fast and can be divided in three main stages: specification, differentiation, and hepatic outgrowth (reviewed in [41]). By 5 dpf, the liver is fully functional and consists of two lobes, with an overall oblong shape [42]. Since hepatotoxic effects are mainly due to metabolic processes, which require a certain time to be executed, experiments were performed at 132 hpf. As the positive control we used paracetamol and ethanol, which have been shown to produce hepatotoxicity in humans and zebrafish [43].

#### 2.6.1. Hepatomegaly and Liver Necrosis Evaluation

**Figure 5:**
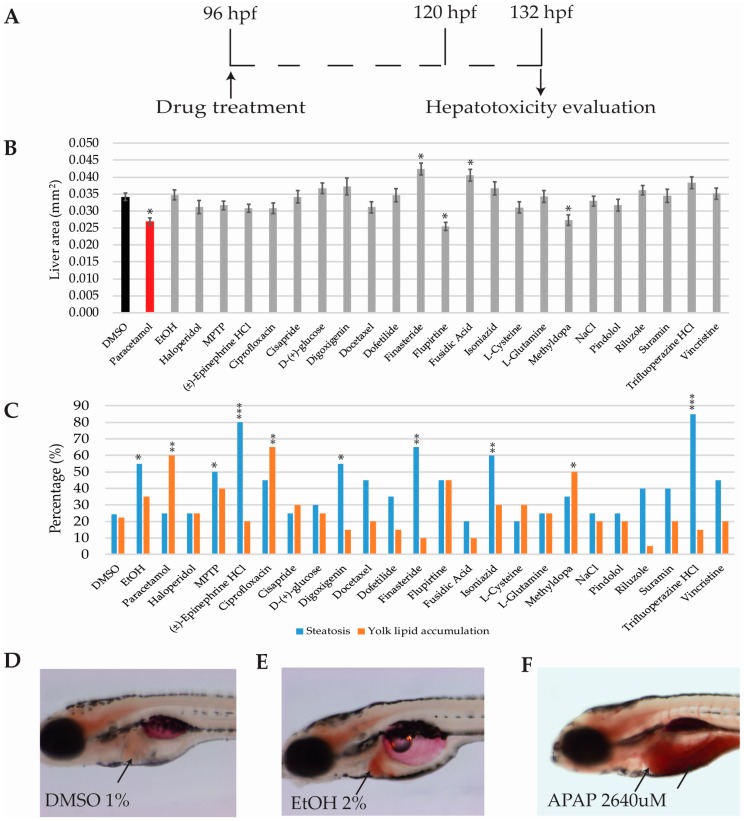
Hepatotoxicity results (**A**) Scheme of the experimental procedure (**B**). Bar graphs showing average liver area in mm. (**C**) Bar graphs showing the percentage of larvae presenting steatosis or yolk lipid accumulation after oil red O stain (**D**–**F**) Representative oil red O whole mount staining images of (**D**) DMSO, (**E**) EtOH and (**F**) APAP; black arrows point at non-affected liver (**D**), liver with steatosis (**E**), and yolk lipid retention (**F**), respectively. Asterisks indicate statistical significance after One-way ANOVA (liver area) or Fisher’s exact test (steatosis and yolk lipid retention): * *p* < 0.05; ** *p* < 0.01; *** *p* < 0.001. Black bar: negative control (**B**). Red bar: positive control (**B**). *n* = 20 but for DMSO *n* = 45.

Zebrafish larvae were fixated and photographed after 36 h of drug incubation (96-132 hpf; Figure 5A). The transgenic zebrafish line *Tg(cmlc2:GFP; fabp10:RFP; ela31:EGFP)* expressed RFP protein in all liver cells. The analysis of fluorescence intensity allowed for the detection of drugs affecting liver size or the number of hepatocytes [44]. Thus, drugs reducing the number of hepatocytes (necrosis) translated into reduced RFP area, whereas drugs increasing liver size (hepatomegalia) corresponded with increased RFP area. In that regard, liver areas of the 24 compounds were analysed and compared with those obtained using the DMSO-only group. Three drugs including paracetamol, flupirtine, and methyldopa showed decreased RFP area signal, whereas finasteride and fusidic acid treatments increased the area of the RFP signal (Figure 5B).

#### 2.6.2. Steatosis and Yolk Lipid Accumulation Evaluation

During the first week of development, the unique source of energy for the zebrafish embryo and larva is the yolk sac. Zebrafish yolk consists of 70% neutral lipid, which is metabolized mainly in the liver [45]. Thus, yolk lipid accumulation can be used as an endpoint for liver function since, if impaired, the yolk metabolism and absorption is delayed, which results in higher lipid retention [46]. On the other hand, drug-induced steatosis (hepatocyte lipid accumulation) is an off-target liver effect which can be used to prioritize compounds for development [47,48]. Hence, drugs affecting lipid metabolism in human hepatocytes might be identified by using zebrafish livers [26,49].

In order to assess drugs producing steatosis and yolk lipid accumulation, and subsequent to the RFP filtered images being acquired, zebrafish larvae were stained with Oil Red O. Larvae and were sorted into steatosis positive or negative, yolk lipid accumulation, or both (see materials and methods). Percentages were calculated for each drug and compared with those obtained in the DMSO-only group (Figure 5D).

Seven out of the 24 drugs were considered to be positive for steatosis (a representative image is displayed in Figure 5E): EtOH (55%), MPTP (50%), (±)-epinephrine HCL (80%), digoxigenin (55%), finasteride (65%), isoniazid (60%), and trifluoperazine HCL (85%). Three drugs were considered to produce yolk lipid retention (a representative image is displayed in Figure 5F): paracetamol (60%), ciprofloxacin (65%), and methyldopa (50%). DMSO percentages for steatosis and yolk lipid accumulation were 24.44% and 22.22%, respectively (Figure 5C,D).

## 3. Discussion

Cardio-, neuro-, and hepatotoxicity are the most relevant organ-toxicities promoting drug attrition during preclinical, clinical, and post market stages [10]. Previous studies have shown the relevance of using zebrafish for predicting the possible impact of drugs in those three organs individually [21,29,50,51,52,53,54,55,56]. However, no previous studies have integrated the analysis of these three organ-toxicities in the same animal; a procedure that reduces animal usage, experimental time and costs, and the quantity of the tested compound.

Results obtained through the ZeGlobalTox assay show high sensitivity, specificity, and accuracy values when we compare the zebrafish experimental data with known human toxicity outputs (Table 3). This is indeed a promising conclusion, given the need for predictive and cost-effective procedures required to narrow down the number of compounds reaching expensive and time-consuming mammalian and clinical studies. Altogether, we propose ZeGlobalTox could be used to reduce the time and costs of drugs for being approved, together with improving 3Rs policies during the whole drug discovery process. Nonetheless, we will discuss below a number of aspects to be considered in order to improve this approach.

Four endpoints were analysed for cardiotoxicity evaluation—BPM, QTc, EJF, and cardiac arrest. A drug was considered cardiotoxic when one of these parameters was found to be statistically different when compared to the DMSO-only group. Our analysis has detected cardiotoxic end-phenotypes in 9 out of 12 human cardiotoxic compounds present in the study. However, from 6 drugs reported to produce QTc prolongation in humans—haloperidol, (±)-epinephrine HCL, ciprofloxacin, cisapride, dofetilide, and trifluoperazine HCL—only haloperidol treated larvae displayed that phenotype. Furthermore, pindolol, not reported to produce QTc prolongation in humans, showed increased QTc in zebrafish larvae. This latter phenotype might be explained by pindolol non-selective blockage of heart ß-receptors. Interestingly, bradycardia was detected in 4 out of 6 drugs producing QTc prolongation in humans. This is consistent with results presented by Wen et al. [57], which showed a correlation between drugs producing QTc prolongation (in dogs) and bradycardia in zebrafish. On the other hand, tachycardia was observed in d-(+)-glucose treated zebrafish larvae. Although glucose is generally innocuous for humans, cardiotoxicity has been reported in hyperglycemic patients and patients suffering from diabetes [58,59,60]. A correlation between high blood glucose levels and poorer outcomes after cardiac arrest has also been described [61]. Therefore, high doses of glucose might also be related to increased cardiotoxicity risk in humans. We hypothesize that tachycardia detected in zebrafish might be due to the need for eliminating/compensating high glucose concentrations as fast as possible. Cardiotoxic false negatives (FN) such as paracetamol, ethanol, and (±)-epinephrine HCL could be explained by differences among human and zebrafish physiology or by the ZeGlobalTox procedure, where cardiotoxic effects are analysed only 4 h after drug incubation. Most cardiotoxic effects can be detected shortly after compound incubation; however, these three compounds might require a longer exposure to reproduce their known cardiotoxic effects. This seems certain for ethanol and paracetamol, since their human cardiotoxic impact is observed as a late effect after drug poisoning. In fact, paracetamol has been reported to have no impact on the heart rate in zebrafish larvae [50,57], to the point that Wen et al. [54] included this drug as a negative cardiotoxic drug [57]. In summary, we support zebrafish as a powerful tool for predicting drug-induced cardiotoxic liabilities in humans, including typical repolarization and depolarization end-phenotypes such as QTc or EJC. However, our experimental methodology—drug exposure timing, chosen drug concentration, image acquisition, and image analysis—might require some further improvement to facilitate a more accurate detection of some of the analysed parameters.

Regarding neurotoxicity assessment, motor behaviour might be affected by neurotoxic, but also by non-neurotoxic compounds that affect the function of the nervous system, such as hypnotic or neuroactive drugs [52,54,60]. This ambivalence could have promoted the identification of a larger percentage of false positives (FP). However, we observed high specificity, since no false positives have been detected. On the other hand, better sensitivity is indeed required because five compounds known to produce some kind of neurotoxicity in humans did not alter larvae locomotion significantly. Thus, we suggest locomotion results should be interpreted cautiously. Indeed, we propose that drugs altering zebrafish locomotion should be tagged with a red-flag, since they could signal a possible Central Side Effect impact. However, drugs not influencing larvae locomotion cannot be tagged safe for neurotoxicity, since they might be neurotoxic without affecting locomotor neural pathways. In that regard, future ZeGlobalTox experimental versions might include a more comprehensive assessment of neural tissue after drug incubation—neuronal mortality, axonal growth defects, etc.

Previous studies have shown the robustness of zebrafish for hepatotoxicity prediction [43,44]. This robustness is supported by a high degree of genetic conservation for the enzymes and pathways required in drug metabolism, such as ARH receptors, CYP enzymes, or Adh isoinzymes, which are present, and functional, from early developmental stages, including our experimental window [62,63,64]. Three phenotypic endpoints were analysed for hepatotoxicity evaluation: liver area, steatosis, and yolk lipid retention. A drug was considered hepatotoxic when at least one of these parameters was statistically different when compared to the DMSO-only group. Consistent with previous studies, we show that paracetamol reduces liver size and increases yolk lipid accumulation. Thus, by reducing the hepatocyte number and/or viability, paracetamol was reducing zebrafish liver size and impairing its function, which led to a decreased lipid metabolism and therefore, its accumulation in the yolk. On the other hand, larvae treated with 2% ethanol showed steatosis but no impact on the liver size or yolk lipid accumulation. Steatosis promoted by 2% ethanol has been extensively reported [26,49,65]. However, there are controversial results regarding the ethanol impact on liver size. Gong et al. [44] identified a reduction in liver size, but Sadler et al. showed hepatomegaly [26,27,49]. In our hands, 2% ethanol did not significantly affect the liver area. However, we detected more rounded livers (shape differences). This phenotype agreed with [26] and might be indicative of an inflammatory process, which later leads to hepatomegaly. Larvae treated with (±)-epinephrine HCL, digoxigenin, and finasteride were found to produce significant higher percentages of hepatic steatosis when compared to the DMSO-only treated group. (±)-epinephrine is not reported to be hepatotoxic in humans. However, it is known that high levels of epinephrine stimulate lipolysis in adipose tissue liberating free fatty acids to the blood, which are then absorbed by the liver that converts them to triglycerides [64,66]. Furthermore, (±)-epinephrine stimulates the breakdown of glycogen in the liver releasing glucose [67]. Glucose can also be converted to fatty acids and finally into triglycerides [68]. Thus, high (±)-epinephrine concentrations might lead to an excessive accumulation of triglycerides in hepatocytes producing, as a side effect, hepatic steatosis in zebrafish larvae. Finasteride and digoxigenin are both extensively metabolized in the liver. Finasteride is a 5-alpha reductase inhibitor that is metabolized via the cytochrome P450 system (CYP 3A4). No severe hepatotoxicity or clinical liver injury has been reported. However, some publications report mild transient serum aminotransferase elevations occurring during finasteride therapy [69]. Digoxigenin is a steroid that when attached to sugars forms glycosides. Digoxigenin is metabolized in the liver via the human liver alcohol dehydrogenase [70]. Thereby, hepatic steatosis, observed after digoxigenin treatment might be originated by a similar mechanism to that seen after ethanol 2% treatment. Consistent with that, in our approach, both treatments cause steatosis in the same percentage (Figure 5C). Finally, regarding MPTP, its hepatotoxicity in humans is not known, however it has been reported to be hepatotoxic in rat livers or isolated hepatocytes [71,72]. Consistent with these studies, steatosis was observed in MPTP treated larvae. All in all, the predictive power of zebrafish hepatotoxicity assessment, may be greater than most in silico or in vitro approaches that are traditionally used [73].

Overall, our results show ZeGlobalTox to be a reliable method to red flag a toxic compound according to its putative general organ liability. On its current methodological version—preliminary AcuteTox, drug concentration, drug exposure timing, and typology of end-phenotypes—it yields an overall high sensitivity, specificity, and accuracy at identifying specific organ toxicities. However, we acknowledge some adjustments need to be implemented to more accurately segment the general organ-toxicities into specific end-phenotypes (i.e., General cardiotoxicity vs. specific QTc prolongation). Moreover, the exposure to NOEC might yield some false negatives. In that sense, testing more than one concentration might provide a better understanding of a possible drug-induced organ liability, if that phenotype requires a higher than NOEC concentration to be triggered. In spite of those possible drawbacks, we expect our results will further support the use of zebrafish as an appropriate model to be exploited in the early phases of drug discovery/development. In that regard, zebrafish could become the chosen model to bridge the gap between low predictive but high throughput in vitro studies and high predictive but expensive and time-consuming in vivo mammalian studies.

## 4. Materials and Methods

### 4.1. Materials and Chemicals

The 20 chemicals used in the present study were chosen and kindly provided by Pivot Park Screening Centre (Oss, The Netherlands) to be tested in a single-blind test: ciprofloxacin, cisapride, l-cysteine, digoxigenin, docetaxel, dofetilide, (±)-epinephrine hydrochloride, finasteride, flupirtine, fusidic acid, d-(+)-glucose, l-glutamine, isoniazid, methyldopa, pindolol, riluzole, sodium chloride (NaCl), suramin, trifluoperazine hydrochloride, and vincristine. The six chemicals used as controls were purchased from Sigma-Aldrich (Sant Louis, MO, USA): dimethyl sulfoxide (DMSO) (D8418), ethanol (EtOH) (02860-1L), haloperidol (H1512), 1-methyl-4-phenyl-1,2,3,6-tetrahydropyridine (MPTP) (M0896-10MG), paracetamol (APAP, acetaminophen Bioxtra) (A7085-100G), and 4-diethylaminobenzaldehyde (DEAB) (D86256).

### 4.2. Zebrafish Maintenance

Zebrafish embryos were obtained by mating adult fish through standard methods. All experiments were performed on zebrafish larvae from 4 dpf until 5.5 dpf, with the exception of the Acute Toxicity test (see “zebrafish exposure conditions” below). Transgenic zebrafish (*Danio rerio*) *Tg(cmlc2:GFP; fabp10:RFP;ela31:EGFP)* was obtained by crossing individual transgenic lines and were kept according to established standard procedures. *Tg(cmlc:GFP)* [50] expresses Green Fluorescent Protein (GFP) in cardiomyocytes, *Tg(fabp10:RFP)* [74] expresses Red Fluorescent Protein (RFP) in hepatocytes, and *Tg(ela31:EGFP)* [75] expresses enhanced Green Fluorescent Protein (EGFP) in pancreatic cells. In the present study, pancreatic toxicity was not analysed, but since it was not affecting the current image analysis, and it might become useful in future experiments, the pancreatic reporter line was kept inside the complete transgene line.

### 4.3. Drug Exposure Conditions

Mortality and Developmental toxicity were assessed through an Acute Toxicity test, adapted from specific OECD guidelines (FET: Fish Embryo Toxicity; OECD 236). Thus, 20 wild type (wt) zebrafish embryos per condition were incubated with tested compounds from 3 to 96 hpf. The test was performed in 5 logarithmic concentrations per drug (from 0.1 µM to 1 mM). Each larva was analysed for mortality, body deformity, edema, tail detachment, pigmentation, heart activity, heart edema, and motor activity. For every compound, a no observed effect concentration (NOEC) was identified to use in the following experiments. The concentrations for drugs used as organo-toxic positive controls were obtained from previous publications and in-house validation: paracetamol (2600 µM; [46]), EtOH (2%; [26]), MPTP (100 µM; [30]), and haloperidol (10 µM; [21]). DMSO 1% was used as the negative control in all experiments.

For the ZeGlobalTox assay, fertilized *Tg(cmlc2:GFP; fabp10:RFP; ela31:EGFP)* zebrafish embryos were collected in E3 medium (5 mM NaCl, 0.17 mM KCl, 0.33 mM CaCl_2_, 0.33 mM, MgSO_4_) in Petri dishes. At 3, 24, and 48 hpf, dishes were observed and all not fertilized, abnormal, or coagulated eggs were discarded. At 96 hpf, 20 larvae per condition were incubated with the NOECs from the different drugs and allowed to develop until 132 hpf at 28.5 °C.

### 4.4. Cardiotoxicity Evaluation in Zebrafish Larvae

**Figure 6:**
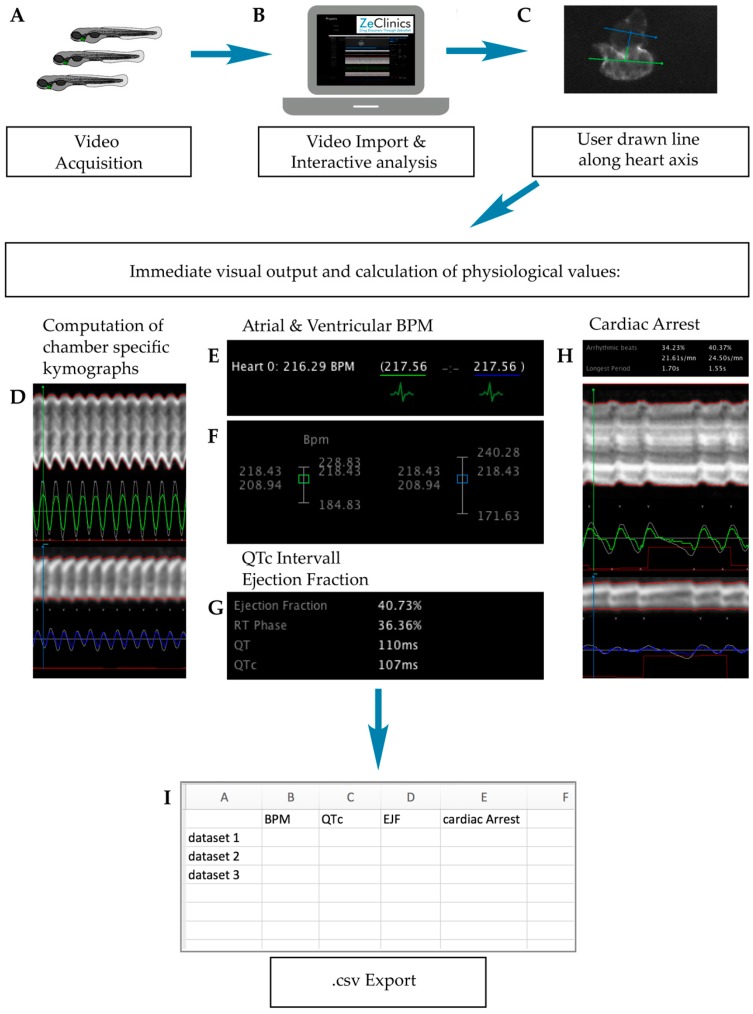
ZeCardio β software user pipeline. (**A**) Video acquisition of larvae incubated with the candidate drug; (**B**) Video import into the software; (**C**) User drawn line along the heart axis; (**D–H**) GUI (Graphical User Interface) display of (**D**) Chamber kymographs; (**E**) atrial and ventricular BPM (Beats Per Minute) values; (**F**) Distribution plot over time of atrial and ventricular BPM; (**G**) QTc interval and EJF (Ejection Fraction) values and (**H**) Cardiac arrest events; (**I**) Output values are presented in .csv format. Kymographs and measurements are displayed in green or blue for ventricle or atrium respectively.

After 4 h of drug incubation (100 hpf), zebrafish larvae were anesthetized by immersion in 0.7 µM tricaine methanesulfonate (A4050, Sigma-Aldrich, Saint Louis, MO, USA)/E3 solution. 10 µM haloperidol treated embryos were used as positive cardiotoxic controls. The 1% DMSO treated embryos were used as negative cardiotoxic controls. Embryos were positioned in an agarose based mold to allow their appropriate orientation under the fluorescence stereo microscope (Olympus MVX10). Individual fluorescent hearts were recorded during 60 s each (Figure 6A). Videos were acquired with a high-speed recording camera (Hamamatsu C11440 ORCA-flash 2.8) and analysed with the ZeCardio^®^ β software to extract different cardiac parameters—heart rate, cardiac arrest, QTc prolongation, and Ejection Fraction (EJF) (Figure 6B).

ZeCardio^®^ β software, developed by ZeClinics and currently in β status, provides a graphical user interface (GUI) that facilitates the semi-automatic analysis of living heart videos. Interactive analysis of the different parameters functions as follows: The user draws a line along the heart axis, from ventricle to atrium, to initiate the calculation. At the ventricle and atrium, an additional line perpendicular to the heart axis (first line) is automatically displayed (Figure 6C). All lines can be subjected to modification of their angles and lengths. From the line selections, two outputs are generated: (i) A kymograph for each of the lines that allows, on one hand, the visual inspection and easy identification/validation of phenotypes (Figure 6D) and, on the other hand, it is used for individual beat detection (Figure 6E); (ii) A numerical output that is displayed in the ZeCardio^®^ GUI.

Heart beat frequency for each chamber is detected and frequencies are presented in the GUI as a mean. A plot distribution is used for assessing beat stability over time (Figure 6F). In the same fashion, chamber specific cardiac arrest is measured as the longest beating pause (Figure 6H). No beating chambers and/or incorrect bpm can be manually flagged when detected.

For calculation of the QTc interval (linearly corrected QT interval), the Framingham formula (QTc = QT + 0.154 (1 − RR)) was adjusted for zebrafish as QTc = QT + 0.154 (2.66 − RR). RR = 6.6 ms/measured bpm is applied (Figure 6G). Finally, the Ejection Fraction, calculated as the maximal dilatation (the diastolic diameter, DD) versus the maximal contraction (systolic diameter, SD) is measured in % as EF% = (DD − SD)/DD*100 (Figure 6G). Computed values for the described parameters were exported in .csv format (Figure 6H).

### 4.5. Neurotoxicity Evaluation in Zebrafish Larvae

Immediately after heart video acquisition, larvae are washed with E3 medium to remove tricaine methanesulfonate from the solution. Fresh drug solution is added and the larvae are transferred individually in a volume of 150 µL to 96 well plates.

Neurotoxicity is analysed at 120 hpf by locomotion assessment using the EthoVision XT 11.5 software and the DanioVision device from Noldus Information Technologies, Wageningen, The Netherlands. This closed system consists of a camera placed above a chamber with circulating water and a temperature sensor set at 28 °C. The 96-well plates are placed in the chamber, which can then be illuminated with white light using the software. Larvae are then left for 20 min under these conditions and with the lights on to help their acclimation. Finally, larvae locomotion is measured during 50′ under the following light/dark conditions: 10′ darkness-10′ Light-10′ darkness-10′ Light-10′ darkness. Total distance moved (in mm) is acquired under this light/dark trial. Due to circadian rhythms, all locomotion assays were performed from 13:00 pm onwards to ensure steady activity of the zebrafish [76].

Neurotoxicity is assessed by comparing locomotion differences among the tested compounds (solved in DMSO) and the negative control group (DMSO 1%-only).

### 4.6. Hepatoxicity Evaluation in Zebrafish

After 36 h of drug incubation (132 hpf), embryos are fixed in 4% paraformaldehyde (158127-500G, Sigma-Aldrich, Saint Louis, MO, USA) for 2–4 h at room temperature (RT) and then are 3× washed with PBS. 

#### 4.6.1. Liver Area Analysis

Fixed larvae are observed under an Olympus MVX10 fluorescent stereo microscope and photographed with a digital camera (Olympus DP71) and the cell’D software. RFP filtered images of the liver were taken and their areas were analysed using the FIJI software for hepatomegaly and necrosis detection.

#### 4.6.2. Oil Red O Staining

**Figure 7:**
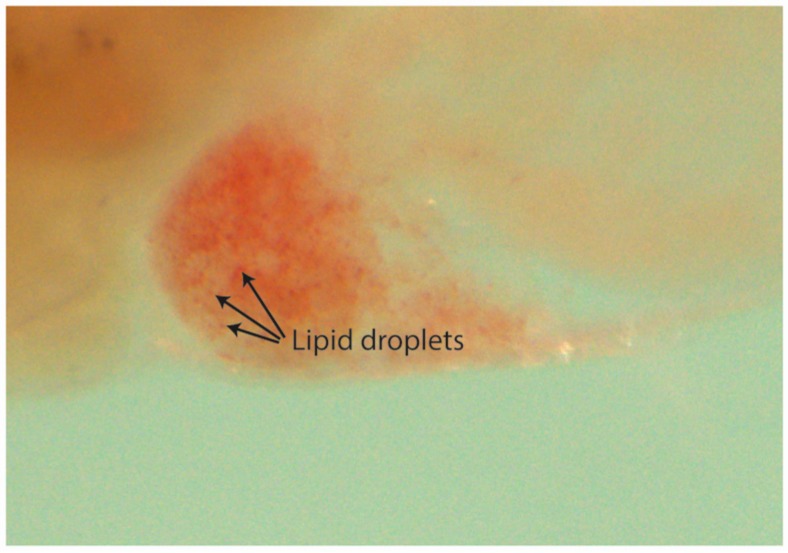
Lipid droplets on a zebrafish liver stained with Oil Red O. Steatosis is considered when three or more droplets are seen within the liver area.

Oil Red O is a lysochrome dye used for the staining of neutral triglycerides and lipids. In order to detect the presence of steatosis and yolk lipid retention, zebrafish larvae were stained with Oil Red O (O0625-25G, Sigma-Aldrich, Saint Louis, MO, USA) as described in [77]. Briefly, the skin pigment from fixed larvae is removed by incubating with bleaching solution (for 10 mL: 6 mL H_2_O, 0.25 mL 20× SSC, 0.5 mL formamide, 3.3 mL H_2_O_2_) during 20 min at RT. Then, the larvae are 5× washed with PBS. Bleached embryos are first submerged in 85% Propylene glycol (PG) (134368-1L, Sigma-Aldrich, Saint Louis, MO, USA) for 10 min and then in 100% PG for another 10 min before staining them with Oil Red O 0.5% in 100% PG (overnight, at RT and with gentle rocking). Oil Red O stained embryos are washed in 100% PG for 30 min, 50 min in 85% PG, and 40 min in 85% PG with an equal volume of PBS. Finally, embryos are washed 1× with PBS before adding 80% glycerol (G7757-500ML, Sigma-Aldrich, Saint Louis, MO, USA). Bright field images are taken to detect both steatosis and yolk lipid accumulation. For steatosis, larvae are considered positive when 3 or more round lipid droplets are visible within the hepatic parenchyma (Figure 5E and Figure 7; [77]). Yolk lipid retention is considered positive when a red strong signal is observed in the yolk area (Figure 5F).

Embryos were incubated with ethanol 2% as the positive control for steatosis [26,49] and with APAP 2600 µM as the positive control for necrosis and yolk lipid accumulation [46]. DMSO 1% treated larvae were used as the negative control group.

### 4.7. Statistical Analysis

Data were analysed using the IBM SPSS Statistics version 20.0 software (Armonk, NY, USA). Data are presented as mean ± standard error (SE). Prior to the analyses, the Shapiro-Wilk test was used to assess the normality of the distribution of the dependent variables. Not normally distributed variables were transformed using Templeton’s two-step method for transforming continuous variables to normal variables [78]. Statistical analysis of the data for the cardiotoxic and neurotoxic parameters as well as for liver size measurements were performed using One-way ANOVA followed by the Dunnett test. Fisher’s exact test was used for data analysis of the steatosis and yolk lipid retention. Results were statistically compared between drug-treated groups and untreated (DMSO) group. Differences were considered statistically significant when *p* < 0.05.

## Figures and Tables

**Table 1:** Selected compounds and their toxicity in humans. White background: tested compounds. Grey background: compounds used as controls. Abbreviations: HCL (hydrochloride), NaCl (sodium chloride), MPTP (1-methyl-4-phenyl-1,2,3,6-tetrahydropyridine).

Drug	Cardiotoxicity	Neurotoxicity	Hepatotoxicity
Acetaminophen	Toxic ^1,2,3^	Toxic ^2,3^	Toxic ^1,2,3^ [24]
Ethanol	Toxic ^1^	Toxic ^1^	Toxic [26,27]
Haloperidol	Toxic ^1,2,3^ [19,20,21]	Toxic ^1,2,3^	Safe
MPTP	*A*	Toxic [22,30,31]	*A*
(±)-Epinephrine HCL	Toxic ^1,2,3^	Toxic ^1,2,3^	Safe
Ciprofloxacin	Toxic ^1,2,3^	Toxic ^1,2,3^	Toxic ^1,2,3^
Cisapride	Toxic ^1,3^	Safe	Safe
d-(+)-glucose	Safe	Safe	Safe
Digoxigenin	Toxic ^1^	Safe	Safe
Docetaxel	Toxic ^2,3^	Toxic ^1,2,3^	Toxic ^1,2,3^
Dofetilide	Toxic ^1,2,3^	Safe	Safe
Finasteride	Safe	Safe	Safe
Flupirtine	Safe	Safe	Toxic ^4^
Fusidic Acid	Safe	Safe	Toxic [32,33]
Isoniazid	Safe	Toxic ^1,2,3^	Toxic ^1,2,3^
l-Cysteine	Safe	Safe	Safe
l-Glutamine	Safe	Safe	Safe
Methyldopa	Safe	Toxic ^1,3^	Toxic ^1,2,3^
NaCl	Safe	Safe	Safe
Pindolol	Toxic ^1,2,3^	Toxic ^2,3^	Safe
Riluzole	Toxic ^2,3^	Safe	Toxic ^2,3^
Suramin	Safe	Toxic [34,35]	Safe
Trifluoperazine HCL	Toxic ^1,2,3^	Toxic ^1,2,3^	Toxic ^1,2,3^
Vincristine	Toxic ^1,2,3^	Toxic ^1,2,3^	Safe

^1^ TOXNET (Hazardous Substances Data Bank, HSDB); ^2^ Side effects (EMBL; European Molecular Biology Laboratory); ^3^ drugs.com; ^4^ ema.europa, *A* effects in humans not known.

**Table 2:** Non Observed Effect Concentration (NOEC), Lowest Observed Effect Concentration (LOEC), and Lethal Concentration 50 (LC50) of selected compounds at 96 hpf. White background: tested compounds. Grey background: DEAB, used as positive toxic control. Abbreviations: NaCl (sodium chloride), DEAB (Diethylaminobenzaldehyde).

Drug	96 hpf NOEC (µM)	96 hpf LOEC (µM)	96 hpf LC50 (µM)
DEAB	1.00	10.00	185.99
(±)-Epinephrine hydrochloride	1000.00	N.A.	3.11 × 10^10^
Ciprofloxacin	1000.00	N.A.	7.01 × 10^9^
Cisapride	1000.00	N.A.	2935.74
d-(+)-glucose	1000.00	N.A.	N.A
Digoxigenin	100.00	1000.00	N.A
Docetaxel	10.00	100.00	N.A
Dofetilide	10.00	100.00	N.A
Finasteride	10.00	100.00	31.62
Flupirtine	10.00	100.00	136.05
Fusidic Acid	10.00	100.00	124.15
Isoniazid	1000.00	N.A.	N.A
l-Cysteine	1000.00	N.A.	N.A
l-Glutamine	1000.00	N.A.	N.A
Methyldopa	1000.00	N.A.	N.A
NaCl	1000.00	N.A.	N.A
Pindolol	100	1000	6608.09
Riluzole	1	10	13.71
Suramin	100	1000	196.56
Trifluoperazine hydrochloride	10	100	31.62
Vincristine	10	100	31.62

**Table 3:** Zebrafish versus Human predictive assessment of Cardiotoxicity, Neurotoxicity, Hepatotoxicity, and ZeGlobalTox. White background: tested compounds. Grey background: Positive toxic controls. Abbreviations: TN: true negative, TP: true positive, FN: false negative, FP: false positive, PPV: positive predictive value, NPV: negative predictive value. Specificity: TN/(TN + FP); sensitivity: TP/(TP + FN); Accuracy: (TP + TN)/(TP + TN + FP + FN); PPV: TP/(TP + FP); NPV: TN/(TN + FN).

Drug	Cardiotoxicity	Neurotoxicity	Hepatotoxicity	
(±)-Epinephrine HCL	FN	TP	FP	
Ciprofloxacin	TP	FN	TP	
Cisapride	TP	TN	TN	
d-(+)-glucose	FP	TN	TN	
Digoxigenin	FN	TN	FP	
Docetaxel	TP	TP	FN	
Dofetilide	TP	TN	TN	
Finasteride	TN	TN	FP	
Flupirtine	TN	TN	TP	
Fusidic Acid	TN	TN	TP	
Isoniazid	TN	FN	TP	
l-Cysteine	TN	TN	TN	
l-Glutamine	TN	TN	TN	
Methyldopa	TN	FN	TP	
NaCl	TN	TN	TN	
Pindolol	TP	TP	TN	
Riluzole	TP	TN	FN	
Suramin	TN	FN	TN	
Trifluoperazine HCL	TP	TP	TP	
Vincristine	TP	TP	TN	
Acetaminophen	FN	TP	TP	
Ethanol	FN	FN	TP	
Haloperidol	TP	FN	TN	
MPTP	-	TP	-	**ZeGlobalTox**
Specificity	90%	100%	77%	89%
Sensitivity	69%	54%	80%	68%
Accuracy	78%	75%	82%	78%
PPV	90%	100%	73%	88%
NPV	69%	65%	83%	72%

## Abbreviations

MDPI	Multidisciplinary Digital Publishing Institute
DOAJ	Directory of open access journals
TLA	Three letter acronym
LD	Linear dichroism
hpf	Hours post fertilization
